# Folate‐Containing Fermented Foods of Plant and Animal Origin: The Value of Traditional Foods to Tackle Folate Deficiency

**DOI:** 10.1002/fsn3.72126

**Published:** 2026-07-22

**Authors:** Rida Khan, Sanaullah Iqbal, L. Suzanne Suggs, Pedro Marques‐Vidal

**Affiliations:** ^1^ Faculty of Communication, Culture and Society Institute of Communication and Public Policy, Universita Della Svizzera Italiana Lugano Switzerland; ^2^ Department of Food Science and Human Nutrition University of Veterinary and Animal Sciences Lahore Pakistan; ^3^ Department of Medicine, Internal Medicine Lausanne University Hospital (CHUV), University of Lausanne Lausanne Switzerland

**Keywords:** biofortification, fermentation, fermented foods, folate, folic acid, traditional foods

## Abstract

Folate is a vital micronutrient involved in many essential biosynthetic pathways in the human body. Increased requirements during pregnancy make folate deficiency a global health concern. Supplemental folic acid prescribed to prevent/correct the deficiency has its limitations, making dietary folate superior to supplementation. This review provides a comprehensive overview of traditional fermented foods worldwide, their folate concentrations, microbial strains, and potential to improve folate status in animal models. Relevant studies investigating the folate content in fermented foods were identified. This review covers a variety of traditional plant‐ and animal‐based fermented foods. Nigerian ogi fermented with *Lactiplantibacillus plantarum* (formerly known as *Lactobacillus plantarum* ) and *Candida tropicalis* had the highest folate among plant‐based foods (3097 μg/100 mL), while yogurt containing *L. plantarum* had the highest folate in animal‐based foods (6323 μg/100 mL), providing ~500%–1000% of the recommended dietary allowance for pregnant women. *L. plantarum* was the most common strain found in foods with high folate concentrations. Seven animal studies demonstrated promising results supporting the effectiveness of fermented foods in improving folate status. This review concludes that traditional fermented foods are a good source of folate, with certain bacteria boosting folate levels, making biofortified fermented products a promising alternative to folic acid supplementation to decrease folate deficiency.

## Graphical abstract

Traditional fermented foods consumed around the world are natural sources of folate. Certain microbes, particularly *Lactiplantibacillus plantarum*, enhance folate production during the fermentation process, making fermented foods a promising dietary approach to improve folate status and help prevent folate deficiency.

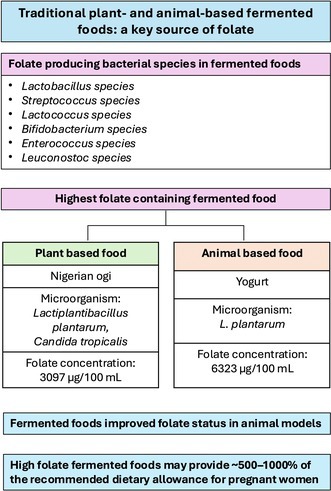

## 1 Introduction

Folate, also known as vitamin B_9_, is a naturally occurring water‐soluble vitamin. It is an important cofactor in nucleotide synthesis and amino acid metabolism. It is essential for deoxyribonucleic acid (DNA) replication, cell fission, and growth, hence important for red blood cell synthesis and maturation (Xiu and Field 2020). Folate, as a coenzyme for thymidylate synthase and dihydrofolate reductase, plays a crucial role in the formation of thymidylate, a vital precursor to DNA and ribonucleic acid (RNA) (Field et al. 2018). Folate cannot be synthesized de novo and must be obtained through adequate dietary or supplemental intake (Iyer and Tomar 2009).

Folate requirements vary throughout the life course, and folate intake is particularly important during periods of rapid growth and development, including infancy, childhood, adolescence, and pregnancy. Among these groups, pregnant women have the highest folate requirements due to increased maternal tissue growth, placental development, and fetal cell proliferation (Lamers 2011). Folate plays a critical role during the first 28 days following conception, a period during which neural tube closure occurs. Consequently, maintaining adequate folate status before conception and throughout pregnancy is widely recommended to support optimal maternal and fetal health (Rísová et al. 2024). The recommended dietary allowance (RDA) of folate is 400 μg for adults, while pregnant and lactating women require 600 and 500 μg, respectively (World Health Organization 2004). Folate deficiency is a major public health concern as it is the most common cause of anemia in women of reproductive age (WRA) (Brittenham et al. 2023). Apart from decreased intake of folate, increased requirements during pregnancy and lactation are frequent causes of deficiency (World Health Organization 2004). This is why it is recommended for women of childbearing age to consume 600 μg folic acid (synthetic form of folate) either from supplements or fortified food in addition to a folate‐rich diet (U.S. Department of Agriculture and U.S. Department of Health and Human Services 2020). Suboptimal maternal intake of folate causes adverse pregnancy outcomes, including miscarriage, anencephaly, and neural tube defects (Wang et al. 2017).

Given the crucial role of folate in maintaining health, it's imperative to address folate deficiency, and potential public health approaches include enhancing dietary intake, supplementation, food fortification, and biofortification (Darnton‐Hill 2019). Among these approaches, food fortification is a cost‐effective, widely accessible, passive intervention strategy that provides continuous population‐level benefits (Osendarp et al. 2018). The benefits of folic acid fortification include a reduction in the incidence of neural tube defects and anemia, a decrease in serum homocysteine levels, and a lower risk of cardiovascular diseases (Ismail et al. 2023). However, the synthetic folic acid used in supplementation and food fortification is different from natural folate, which may influence their metabolism, bioavailability, and physiological effects (Scaglione and Panzavolta 2014; Watson and Preedy 2012). Additionally, fortification is only practically feasible in communities consuming processed foods, while in low and middle‐income countries, most foods are prepared at home, where fortification is not possible. Folate occurs naturally in foods in reduced forms such as tetrahydrofolate and its derivatives, whereas folic acid (pteroylmonoglutamic acid) is a synthetic, fully oxidized form that does not occur naturally in foods (Caudill 2010). Folic acid is approximately 1.7 times more bioavailable than natural folate because about 90% of folate in natural foods is present in the form of polyglutamates and requires hydrolysis by folate conjugase enzymes located on the brush border membrane of the small intestine for its conversion to monoglutamates for absorption, while folic acid is already present as a monoglutamate (Institute of Medicine (US) Standing Committee on the Scientific Evaluation of Dietary Reference Intakes and its Panel on Folate, Other B Vitamins, and Choline 2000). However, after absorption, folic acid is biologically inactive and must undergo sequential reduction by the enzyme dihydrofolate reductase (DHFR) to form dihydrofolate and subsequently tetrahydrofolate, which can then participate in one‐carbon metabolism, while naturally reduced folate requires fewer metabolic conversions before entering cellular pathways (Institute of Medicine (US) Standing Committee on the Scientific Evaluation of Dietary Reference Intakes and its Panel on Folate, Other B Vitamins, and Choline 2000).

While folic acid supplementation helps prevent deficiency in individuals with low dietary intake, excessive intake may pose risks. Human DHFR activity is relatively low and highly variable among individuals; therefore, excessive folic acid intake may exceed the metabolic capacity of the liver, leading to unmetabolized folic acid in the circulation (Bailey and Ayling 2009). Although the clinical significance of circulating unmetabolized folic acid remains under investigation, concerns have been raised regarding its potential effects on the immune system, epigenetic regulation, and disease risk (Fardous and Heydari 2023). Folic acid supplementation can also mask the early signs of vitamin B_12_ deficiency, increasing the risk of irreversible neurological damage (Cuskelly et al. 2007). Additionally, a meta‐analysis of 10 randomized controlled trials revealed a borderline significant increase in overall cancer incidence among individuals who received folic acid supplements compared to control groups (Wien et al. 2012).

These limitations associated with folic acid supplementation call for alternative approaches to tackle folate deficiency. Fermented foods have emerged as a promising dietary source, with several studies demonstrating substantial amounts of folate in them (Cuamatzin‐Garcia et al. 2022). Unlike supplementation and food fortification, which rely on synthetic folic acid, microbes produce naturally occurring folate during fermentation (Rossi et al. 2011). Folate‐producing microorganisms synthesize folates intracellularly and most of it remains within the microbial cells in polyglutamated forms, while only a small proportion is released into the surrounding food matrix. The extracellular folate fraction is typically present as monoglutamate derivatives, which are more readily available for intestinal absorption (Revuelta et al. 2018; Sybesma et al. 2003).

Given their widespread consumption across diverse cultures and regions, fermented foods offer an accessible, sustainable, and culturally acceptable means of enhancing folate intake (Bationo et al. 2020). Naturally fermented foods not only serve as a direct source of folate but also harbor beneficial folate‐producing microbial strains (Bationo et al. 2025). In situ biofortification through fermentation using folate‐producing microorganisms is also a sustainable method to increase dietary folate intake levels (Tamene et al. 2022). Ignoring the impact of fermentation on traditional foods can lead to a significant underestimation of folate adequacy, particularly in populations where fermented foods are dietary staples (Verger et al. 2025). Therefore, this review provides a comprehensive overview of folate concentrations and the microbial strains present in traditional fermented foods of both plant and animal origin that are commonly consumed across various regions of the world. It also explores the folate‐producing capacity of different microbial strains involved in fermentation. Given the increased folate requirements among pregnant women, the folate contribution of these foods is discussed in relation to the RDA for pregnant women. Finally, the review summarizes the available evidence from animal studies on the effectiveness of different fermented foods in improving folate status.

## 2 Methods

A literature search was conducted on PubMed in March 2025 using the search query: (“ferment*”[Title/Abstract] OR “fermented food*”[Title/Abstract] OR “fermentation”[Title/Abstract]) AND (“folate”[Title/Abstract] OR “folic acid”[Title/Abstract] OR “vitamin B_9_”[Title/Abstract] OR “vitamin B 9”[Title/Abstract]). The search yielded 272 articles. Titles and abstracts were initially screened for relevance, followed by a full‐text screening. In addition, relevant articles were identified through citation tracking and reference lists of the initially included studies.

Studies were included if they were written in English, published in peer‐reviewed journals, investigated fermented foods or fermentation processes, and reported on either folate concentration or folate‐producing microorganisms. No restrictions were placed on the publication date. Eligible studies were categorized into the following groups: plant origin fermented foods (*n* = 26), animal origin fermented foods (*n* = 18), and animal studies (*n* = 7). Folate‐producing bacterial species were identified across included studies, with an additional 22 articles specifically reporting on such microorganisms.

Data extracted from studies on fermented foods included product name, country of origin, microbial culture, fermentation or incubation conditions, analytical methods of folate determination and the amount of folate reported. Folate concentrations were standardized to per 100 g or 100 mL units to enable comparison across studies. Based on the reported folate concentrations, the percentage of the RDA for pregnant women (600 μg) (World Health Organization 2004) provided by 100 g or 100 mL of the fermented food was calculated. When folate concentrations were reported on both fresh weight and dry weight basis, %RDA was calculated using the fresh weight concentrations. The %RDA was not calculated for studies reporting folate production by bacteria and was only determined when folate concentration in food was reported (Tables 2 and 3). For studies reporting folate‐producing microorganisms, bacterial species were classified into their respective taxonomic families to enable comparison across genera (Table 1). For animal studies, the extracted data included the source of folate, administered dose, experimental model used, duration of the intervention, and key findings on the physiological outcomes (Table 4).

## 3 Results

### 3.1 Folate‐Producing Bacterial Species

Several bacterial species capable of producing folate have been identified, with LABs being among the most prominent (Table 1). Within this group, the Lactobacillaceae family is the most frequently reported folate producer (Ashagrie et al. 2023; Misci et al. 2021), and within this family, *L. plantarum* has the maximum folate‐producing capacity in yogurt (6323 μg/100 mL) (Wu et al. 2017). The genomic studies also confirm that the plasmids of *L. plantarum* have the gene clusters for the synthesis of folate (Li et al. 2016; Wegkamp et al. 2010). Compared to other LAB species, *L. plantarum* generally possesses a more complete genetic profile for folate biosynthesis (Zhang et al. 2020). *L. delbrueckii* subsp. *bulgaricus* is also known for its folate production (Bozzetti et al. 2025; Laiño et al. 2013; Laiño et al. 2012) and contains multiple genes (*folA*, *folC*, *folP*, and *phoA*) responsible for folate biosynthesis (Laiño, Hebert, et al. 2015). Several other *Lactobacilli*, including *L. fermentum* (Albuquerque et al. 2017; Bationo et al. 2019; Hugenschmidt et al. 2010; Odumosu et al. 2023), *L. rhamnosus* (Albuquerque et al. 2017; Laiño et al. 2014; Wu et al. 2017), *Levilactobacillus brevis* (formerly known as *Lactobacillus brevis* ) (Hugenschmidt et al. 2010), *L. sakei* (Carrizo et al. 2016; Jiménez et al. 2018; Liu et al. 2022; Mosso et al. 2018), *L. reuteri* (Albuquerque et al. 2017; Hugenschmidt et al. 2010), *L. acidophilus* (Albuquerque et al. 2017; Laiño et al. 2014; Wu et al. 2017), *L. helveticus* (Ahire et al. 2013), *L. casei* (Wu et al. 2017), *L. amylovorus* (Laiño et al. 2014), and *L. pentosus* (Salvucci et al. 2016) demonstrate the ability to produce folate. Genetic modification has also enabled folate production in previously non‐producing strains. Wegkamp et al. (2004) transformed a folate‐consuming strain of *L. gasseri* into a producer (7.5 μg/100 mL) using a gene cluster from *Lc. lactis*. Similarly, Santos et al. (2008) introduced a plasmid from *L. plantarum* WCFS1 into *L. reuteri* JCM1112, achieving a 100‐fold increase in folate biosynthesis.

**TABLE 1:** Bacterial species capable of producing folate.

Bacterial species	References
*Lactobacillus* species	
*Lactiplantibacillus plantarum*	Greppi et al. (2017), Li et al. (2016), Wegkamp et al. (2010), Marco et al. (2009), Divya et al. (2012), Hugenschmidt et al. (2010), Salvucci et al. (2016), Carrizo et al. (2017), Purwandhani et al. (2018), Zhang et al. (2020), Khalili et al. (2019), Wu et al. (2017), Tamene et al. (2023), Tamene et al. (2022), Tamene, Baye, et al. (2019), Ashagrie et al. (2025), Bationo et al. (2019), Liu et al. (2019), Carrizo et al. (2020), Carrizo et al. (2016), Rodrigo‐Torres et al. (2019); Elizaquivel et al. (2015), Thompson et al. (2020)
*Limosilactobacillus fermentum*	Greppi et al. (2017), Albuquerque et al. (2017), Hugenschmidt et al. (2010), Bationo et al. (2019), Odumosu et al. (2023)
*Lactobacillus amylovorus*	Laiño et al. (2014)
*Limosilaactobacillus reuteri*	Albuquerque et al. (2017), Hugenschmidt et al. (2010)
*Lactobacillus acidophilus*	Albuquerque et al. (2017), Laiño et al. (2014), Wu et al. (2017)
*Lacticaseibacillus rhamnosus*	Albuquerque et al. (2017), Panda et al. (2018), Carrizo et al. (2016)
*Lactobacillus delbrueckii subsp. bulgaricus*	Laiño et al. (2012), Khalili et al. (2019), Bozzetti et al. (2025)
*Levilactobacillus brevis*	Hugenschmidt et al. (2010)
*Lactobacillus sakei*	Jiménez et al. (2018), Carrizo et al. (2016), Mosso et al. (2018), Liu et al. (2022)
*Lactobacillus pentosus*	Salvucci et al. (2016)
*Lactobacillus helveticus*	Ahire et al. (2013)
*Lacticaseibacillus casei*	Wu et al. (2017)
*Streptococcus* species	
*Streptococcus thermophilus*	Albuquerque et al. (2017), Tomar et al. (2009), Laiño et al. (2012), Laiño, Zelaya, et al. (2015), Da Silva et al. (2016), Zhang et al. (2020), Khalili et al. (2019), Padalino et al. (2012), Cucick et al. (2024), Bozzetti et al. (2025)
*Streptococcus macedonicus*	Laiño, Zelaya, et al. (2015), Laiño et al. (2019)
*Lactococcus* species	
*Lactococcus lactis*	Wegkamp et al. (2007), Da Silva et al. (2016), Zhang et al. (2020), Divya et al. (2012), Divya and Nampoothiri (2015b), Jiao et al. (2020), Gangadharan and Nampoothiri (2011)
*Lactococcus hircilactis*	Tidona et al. (2018)
*Bifidobacterium* species	
*Bifidobacterium adolescentis*	Pompei et al. (2007), Sugahara et al. (2015)
*Bifidobacterium pseudocatenulatum*	D'Aimmo et al. (2012), Sugahara et al. (2015)
*Bifidobacterium catenulatum*	Pompei et al. (2007), D'Aimmo et al. (2012), Sugahara et al. (2015), Padalino et al. (2012)
*Bifidobacterium animalis*	Pompei et al. (2007), Crittenden et al. (2003)
*Bifidobacterium bifidum*	Pompei et al. (2007)
*Bifidobacterium dentium*	Pompei et al. (2007), D'Aimmo et al. (2014)
*Bifidobacterium infantis*	Pompei et al. (2007), Cucick et al. (2024)
*Bifidobacterium longum*	Pompei et al. (2007), Sugahara et al. (2015), Lin and Young (2000)
*Enterococcus* species	
*Enterococcus faecium*	Divya et al. (2012)
*Enterococcus mundtii*	Salvucci et al. (2016), Nawaz et al. (2019)
*Leuconostoc* species	
*Leuconostoc mesenteroides*	Carrizo et al. (2017), Jiménez et al. (2018)

*S. thermophilus* is commonly used as a starter culture in the preparation of fermented dairy products and is also known for its folate‐producing capacity (Bozzetti et al. 2025; Cucick et al. 2024; Khalili et al. 2019; Padalino et al. 2012; Zhang et al. 2020), partly through *folK* and *folP* gene expression (Meucci et al. 2018). Recently, Bozzetti et al. (2025) screened 26 strains of *S. thermophilus* and all produced folate, the concentration of which ranged from 30.9 μg/100 mL to 63.9 μg/100 mL of milk. Another species from the family of *Streptococcaceae*, *S. macedonicus* CRL415, is also known for folate production (Laiño et al. 2019).

Another important folate‐producing LAB is *Lactococcus* (Divya and Nampoothiri 2015b; Divya et al. 2012; Gangadharan and Nampoothiri 2011; Jiao et al. 2020). *Lc. lactis* carries *pabA*, *pabB*, and *pabC* genes for para‐aminobenzoic acid (pABA) production (Wegkamp et al. 2007) and multiple copies of *aeroD*, *aeroE*, *folA*, and *folB* genes for folate production (Zhang et al. 2020). Overexpression of the folKE alone was found to triple folate production, while co‐expression with folC improved folate retention (Sybesma et al. 2003). *Lc. hircilactis* increased the folate content fourfold as a starter culture (Tidona et al. 2018).

A major genus of probiotics, *Bifidobacteria*, produces folate both in the intestine and in the food (Cucick et al. 2024; Padalino et al. 2012). *B. catenulatum* ATCC 27539 was found to produce the highest amount of folate (9295 μg/100 g) (D'Aimmo et al. 2012). Among 17 bifidobacteria of human origin, six strains, including *B. animalis* , *B. bifidum* , *B. catenulatum* , *Bifidobacterium dentium* , *B. infantis* , and *B. longum* , produced folate (Pompei et al. 2007). Almost all human‐residential bifidobacteria can synthesize folate in vitro (Sugahara et al. 2015), though evidence on *B. breve* remains mixed (D'Aimmo et al. 2012; Pompei et al. 2007). *B. adolescentis* MB 239 also shows folate‐producing capacity (Pompei et al. 2007).

Members of the Enterococcaceae family, *Enterococcus faecium* (Divya et al. 2012) and *E. mundtii* (Nawaz et al. 2019; Salvucci et al. 2016) demonstrated folate production. Genetic remodeling/metabolic engineering of folate‐consuming strains has yielded promising results. Among *Leuconostocaceae*, *Ln. mesenteroides* has been identified as a folate producer (Carrizo et al. 2017; Jiménez et al. 2018).

### 3.2 Folate in Fermented Foods

Several foods of both animal and plant origin contain significantly more folate when fermented compared to their non‐fermented forms. Each fermented food hosts unique microbial strains that play a crucial role in the fermentation process. In addition to specific microbial strains, incubation time and temperature are key factors affecting folate biosynthesis. This section discusses popular fermented foods consumed worldwide, their microbial cultures, incubation conditions, and resulting folate levels. Among studies investigating fermented foods of plant and animal origin, folate concentration was mostly determined using microbiological assays (*n* = 33), followed by high‐performance liquid chromatography (HPLC) (*n* = 7). Spectrophotometry was used in two studies (*n* = 2), while liquid chromatography–tandem mass spectrometry (LC–MS/MS) was applied in one study (*n* = 1) (Tables 2 and 3).

**TABLE 2:** Folate‐containing fermented foods of plant origin.

Food product	Origin	Microbial culture	Fermentation/incubation conditions	Folate determination method	Amount of folate (/100 g or mL)	%RDA^a^	References
Traditional fermented foods in Africa							
Tef injera (fermented flat bread made from *Eragrostis tef* )	Ethiopia		Back‐slopping fermentation at room temperature for 3–4 days	Microbiological assay	14.8 μg FW, 39.0 μg/100 g DW	2.38%	Tamene, Kariluoto, et al. (2019)
Injera (fermented flat bread made from tef, sorghum, wheat, and barley)	Ethiopia	*L. plantarum* P2R3FA (isolated from fermented tef batter)	Back‐slopping fermentation at room temperature for 4 days	Microbiological assay	Barley injera: 13.1 ± 0.6 μg FW, 35.4 ± 1.3 μg DW Wheat and sorghum blend injera: 15.4 ± 0.6 FW, 32.7 ± 1.5 μg DW	Barley injera: 2.19%; Wheat and sorghum blend injera: 2.58%	Tamene et al. (2023)
Tef injera (fermented flat bread made from *Eragrostis tef* )	Ethiopia	*L. plantarum* P2R3FA (isolated from fermented tef batter) *S. cerevisiae*	Back‐slopping fermentation at room temperature for 4 days	Microbiological assay	45.3 ± 1.7 μg FW, 131 ± 15 μg DW	7.55%	Tamene et al. (2022)
Tef injera (fermented flat bread made from *Eragrostis tef* )	Ethiopia	*L. plantarum* P2R3FA	Back slopping fermentation 25°C for 3–4 days	Microbiological assay	139.1 ± 6.5 μg DW		Ashagrie et al. (2025)
Tef injera (fermented flat bread made from *Eragrostis tef* )	Ethiopia	LAB (predominantly *L. plantarum* ), highest folate‐producing strain *L. plantarum* P2R3FA	37°C for 24 h	Microbiological assay	0.1–4.3 μg		Tamene, Baye, et al. (2019)
Injera	Ethiopia	*Lactobacillaceae*	Back‐slopping fermentation at room temperature for 3–4 days	Microbiological assay	21.2 μg FW, 60.3 ± 6.9 μg DW	3.53%	Ashagrie et al. (2023)
Ben‐saalga (fermented porridge made from pearl millet)	Burkina Faso	*L. fermentum* , *L. plantarum*	37°C for 24 h Folate‐free medium	Microbiological assay	*L. fermentum* : 2.9 μg *L. plantarum* : 4.4 μg		Greppi et al. (2017)
Ben‐saalga (fermented porridge made from pearl millet)	Burkina Faso	*L. plantarum*, *L. fermentum*	Spontaneous fermentation; Back slopping fermentation at 30°C for 24 h	Microbiological assay	Spontaneous fermentation: 7.1–7.3 μg FW Back slopping: 6.1 μg FW	Spontaneous fermentation: 1.18%–1.22% Back‐slopping: 1.02%	Bationo et al. (2019)
Ben‐saalga (fermented porridge made from pearl millet)	Burkina Faso		Room temperature 10–24 h	Microbiological assay	1.9–2.9 μgf FW	0.32%–0.48%	Bationo et al. (2020)
Ben‐saalga (fermented porridge made from pearl millet)	Burkina Faso	Aerobic mesophilic bacteria, LAB	Spontaneous fermentation at 30°C for 12 h Back slopping step ≤ 37°C for 12 h	Microbiological assay	Spontaneous fermentation: 2.2 FW Back slopping: 1.5 FW	Spontaneous fermentation: 0.37% Back slopping: 0.25%	Saubade et al. (2018)
Fermented pumpkin leaves (*Cucurbita* sp.)	Kenya	*Lactobacillaceae*	Air dried: 65°C for 72 h; Thermal treated: in boiling water for 30 min	LC–MS/MS method	Air dried: 211.1 ± 8.4 μg DW Thermal treated: 269.9 ± 8.3 μg DW	Air dried: 36.85% Thermal treated: 44.98%	Misci et al. (2021)
Ogi (fermented maize cereals)	Nigeria	*L. plantarum, C* *. tropicalis*	24–72 h	Spectrophotometry	3097 ± 37 μg at 24 h of fermentation	516.17%	Okoroafor et al. (2019)
Fermented locust beans, uncooked fermented cassava, cooked cassava (fufu) and cheese	Nigeria	*L. fermentum* MT903311	37°C for 48 h	HPLC	24,105 μg		Odumosu et al. (2023)
Fermented maize‐based porridge, called *Togwa*	Tanzanian	*C. glabrata* TY26	30°C for 46 h	HPLC	6.9 ± 0.1 μg	1.15%	Hjortmo et al. (2008)
Fermented oat and barley cereal		*S. cerevisiae* ALKO743 *C. milleri* ABM4949 (Isolated from oat and rye)	28°C for 24 h	Microbiological assay	12.0 μg	2.00%	Kariluoto et al. (2014)
Traditional fermented foods in Latin America							
Tocosh (fermented potatoes)	Central Peruvian Andes	*Ln. mesenteroides*, *L. sakei*	In running water for up to 12 months	Microbiological assay	*Ln. mesenteroides*: 7.7–10.2 μg *L. sakei* : 7.2–10.0 μg		Jiménez et al. (2018)
Tocosh (fermented potatoes)	Central Peruvian Andes	*L. sakei*	37°C for 24 h	Microbiological assay	76.0–148.4 μg	12.67%–24.73%	Mosso et al. (2018)
Quinoa sourdough	Andean region of South America	*L. plantarum, L. rhamnosus, L. sakei*	30°C for 24 h	Microbiological assay	*L. plantarum* : 14.3 ± 0.6 μg		Carrizo et al. (2016)
Amaranth sourdough		*L. plantarum*	Spontaneous fermentation	Microbiological assay	13.8 ± 0.8 μg		Carrizo et al. (2017)
Fermented pasta from quinoa sourdough	Argentina	*L. plantarum*	30°C for 24 h	Microbiological assay	130.0 ± 10.0 μg dough	26.67%	Carrizo et al. (2020)
Chicha (fermented mazie‐based beverage)	Northwestern Argentina	*L. plantarum*	30°C for 48 h	Microbiological assay	3.0–5.5 μg		Rodrigo‐Torres et al. (2019); Elizaquivel et al. (2015)
Traditional fermented foods in Asia							
Douchi (fermented soybean)	China	*L. plantarum*	37°C for 60 h	HPLC	1.4–1.6 μg at 24 h of fermentation		Liu et al. (2019)
Novel fermented foods of plant origin							
Fermented cucumber and watermelon juice	India	*Lc. lactis* subsp. *cremoris*	37°C for 8 h	Microbiological assay	Fermented cucumber juice: 6.0 ± 0.2 μg Fermented watermelon juice: 2.6 ± 0.2 μg	Fermented cucumber juice: 1.00% Fermented watermelon juice: 0.43%	Gangadharan and Nampoothiri (2011)
Fermented cauliflower‐white bean mixture		*L. plantarum*	30°C for 44 h	Microbiological assay	58.8 ± 2.0 μg FW	9.80%	Thompson et al. (2020)
Fermented soymilk (with passion fruit by‐products)		*S. thermophilus* *L. acidophilus* , *L. reuteri* , *L. fermentum* , *L. rhamnosus*	37°C for 24 h	Microbiological assay	*S. thermophilus* , 132.5 ± 7.7 μg	22.08%	Albuquerque et al. (2017)

Abbreviations: DW, dry weight; FW, fresh weight.

^a^ Percentage of Recommended Dietary Allowance (%RDA) of folate for pregnant women provided by 100 mL or 100 g of food, based on WHO guidelines (World Health Organization 2004).

**TABLE 3:** Folate‐containing fermented foods of animal origin.

Food product	Origin	Microbial culture	Incubation conditions	Folate determination method	Amount of folate (μg/100 g or mL)	%RDA^a^	References
Traditional fermented foods in Asia							
Dahi, fermented milk product	Pakistan	*E. mundtii*	37°C for 24–48 h	Spectrophotometry	~2000–3000		Nawaz et al. (2019)
Dadih, fermented buffalo milk	Indonesia	*L. plantarum*	Room temp. for 48 h	Microbiological assay	2.9 ± 0.4	0.49%	Purwandhani et al. (2018)
Thai fermented fish (plaasom fug)	Thailand	*W. cibaria*	37°C for 48 h	Microbiological assay	414		Deatraksa et al. (2018)
Yogurt	Tibet	*S. thermophilus* , *L. bulgaricus* , *L. plantarum* (isolated from Tibet kefir)	42°C for 6 h	HPLC	Extracellular folate: 372 Intracellular folate: 1202	Extracellular folate: 62.00% Intracellular folate: 200.33%	Zhang et al. (2020)
Traditional fermented foods in Latin America							
Argentinian yogurt	Argentina	*L. delbrueckii subsp. Bulgaricus*, *S. thermophilus*	37°C for 24 h	Microbiological assay	2.2–13.5	0.37%–2.25%	Laiño et al. (2012)
Yogurt	Argentina	*L. delbrueckii subsp. Bulgaricus*, *S. thermophilus*	42°C for 6 h	Microbiological assay	18.0 ± 1.0	3.00%	Laiño et al. (2013)
Fermented milk	Argentina	*L. amylovorus* , *S. thermophilus* , *L. bulgaricus*	42°C for 24 h	Microbiological assay	26.3 ± 0.2	4.39%	Laiño et al. (2014)
Fermented milk	Argentina	*S. thermophilus* , *S. macedonicus* , *L. bulgaricus*	42°C for 6 h	Microbiological assay	18.7 ± 0.7	3.12%	Laiño, Zelaya, et al. (2015)
Fermented milk	Argentina	*S. gallolyticus subsp. macedonicus*	42°C for 24 h	Microbiological assay	4.3 ± 0.3	0.72%	Laiño et al. (2019)
Fermented goat milk	Brazil	*S. thermophilus* , *Lc. lactis* (isolated from goat milk and cheese in Brazil)	37°C for 24 h	Microbiological assay	9.2–31.3	1.53%–5.22%	Da Silva et al. (2016)
Novel fermented foods of animal origin							
Fermented skim milk	India	*Lc. lactis* CM28 (isolated from raw cow milk)	37°C for 8 h	Microbiological assay	6.1 ± 1.3	1.02%	Divya and Nampoothiri (2015b)
Skim milk and ice cream	India	Encapsulated *Lc. lactis* CM22, *Lc. lactis* CM28 (isolated from cow milk)	37°C for 15 h	Microbiological assay	Skim milk *Lc. lactis* CM22: 14.4 ± 0.2 *Lc. lactis* CM28: 11 ± 0.2 Ice cream *Lc. lactis* CM22: 17.4 ± 0.1 *Lc. lactis* CM28: 17.2 ± 0.3	Skim milk *Lc. lactis* CM22: 2.41% *Lc. lactis* CM28: 1.84% Ice cream *Lc. lactis* CM22: 2.90% *Lc. lactis* CM28: 2.87%	Divya and Nampoothiri (2015a)
Fermented milk	Italy	*Lc. hircilactis*, *Lc. laudensis* (isolated from goat and cow milk)	30°C for 48 h	Microbiological assay	*Lc. hircilactis*: 1.6 ± 0.1 *Lc. laudensis*: 0.1 ± 0.2	*Lc. hircilactis*: 0.27% *Lc. laudensis*: 0.01%	Tidona et al. (2018)
		*Lc. lactis* KLDS4.0325	37°C for 24 h	Microbiological assay	60.0	10.00%	Jiao et al. (2020)
Yogurt		*L. plantarum* , *L. casei* , *L. acidophilus*	37°C for 18 h	HPLC	*L. plantarum* : 6323 *L. casei* : 4541 *L. acidophilus* : 4278	*L. plantarum* : 1053.83% *L. casei* : 756.83% *L. acidophilus* : 713.00%	Wu et al. (2017)
		*L. bulgaricus* , *S. thermophilus* , *L. plantarum*	42°C ± 0.2°C for 6 h	HPLC	148.7 ± 9.6	24.78%	Khalili et al. (2019)
Fermented milk		*B. catenulatum* , *S. thermophilus*	37°C for 6 h and 10 h	HPLC	*B. catenulatum* : 28.8 ± 2.0 *S. thermophilus* : 19.0 ± 2.0	*B. catenulatum* : 4.80% *S. thermophilus* : 3.17%	Padalino et al. (2012)
Fermented whey beverage with grape by‐products		*B. infantis* BB‐02, *S. thermophilus* TH‐4	37°C for 24 h	Microbiological assay	40.7	6.78%	Cucick et al. (2024)

^a^ Percentage of Recommended Daily Allowance (%RDA) of folate for pregnant women provided by 100 mL or 100 g of food, based on WHO guidelines (World Health Organization 2004).

#### 3.2.1 Fermented Foods of Plant Origin

**FIGURE 1:**
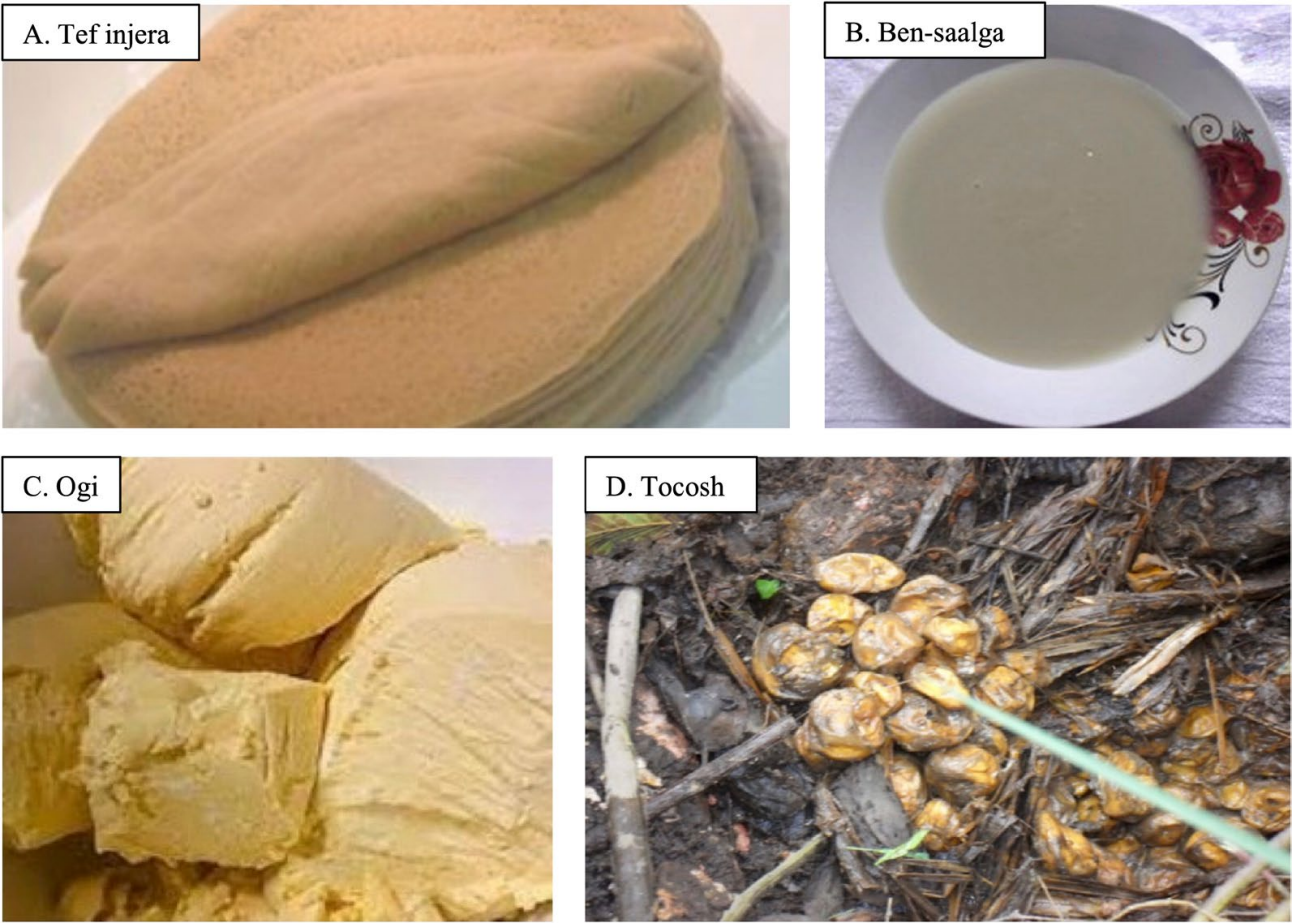
Fermented foods of plant origin: (A) Tef injera [adapted from (Mengesha et al. 2022)], (B) Ben‐saalga [adapted from (Bationo et al. 2020)], (C) Ogi [adapted from (Ndudi et al. 2024)], (D) Tocosh [adapted from (Jiménez et al. 2018)].

Around the globe, people consume various fermented grains and cereal‐based products (Table 2; Figure 1). Pickling and fermenting vegetables and fruits have also been an integral practice of food preservation since ancient times. It not only provides several gut‐friendly prebiotics and probiotics but also adds variety to the meal (Cuamatzin‐Garcia et al. 2022).

##### 3.2.1.1 Traditional Fermented Foods in Africa

In Ethiopia, *Injera* is commonly consumed as a staple food, which is a leavened flatbread made from different cereals (Figure 1A; Tamene et al. 2023). Although Tef injera is the most common one and is made from the ancient cereal crop *Eragrostis tef* (Tamene, Kariluoto, et al. 2019), several other cereals like sorghum, wheat, and barley have been used in the injera preparation in Ethiopian highlands (Tamene et al. 2023; Table 2). The traditional process of making injera involves using leftovers from the previous spontaneous fermentation in the back‐slopping fermentation, where a small part of the spontaneous fermentation product, after ensuring batch quality, is added to the next batch when the temperature drops below 37°C (Saubade et al. 2018). The average folate concentration of injera was 39 μg/100 g, which is significantly lower (*p* < 0.05) than that of the flour (59 ± 11 μg/100 g) (Tamene, Kariluoto, et al. 2019) (Table 2). This study showed that tef flour is a good source of folate and the fermentation increased folate content in the dough in some cases, while baking led to a reduction in folate content (Tamene, Kariluoto, et al. 2019). Tamene et al. (2023) compared the folate content of injera prepared from different cereals and found that the highest folate content (15.4 μg/100 g) was observed in *Lactiplantibacillus plantarum* (formerly known as *Lactobacillus plantarum* ) fermented wheat and sorghum blend injera (3:1) and that its consumption contributed up to 8% of the RDA of folate for WRA. In a similar study, tef injera was prepared using *L. plantarum* P2R3FA previously isolated from fermented tef batter and commercially available *Saccharomyces cerevisiae* (Tamene et al. 2022). The combination of both microorganisms produced higher levels of folate, contributing to 23% of the RDA for WRA (Tamene et al. 2022). In a recent study, Ashagrie et al. (2025) also prepared injera using a high folate‐producing strain, *L. plantarum* P2R3FA. The folate content of injera prepared traditionally was 119.7 ± 0.1 μg/100 g and increased to 139.1 ± 6.5 μg/100 g with *L. plantarum* P2R3FA.

In West Africa, ben‐saalga is a popular traditional fermented pearl millet‐based porridge (Figure 1B). Several studies have been done to examine the microbiological and nutritional composition of ben‐saalga (Bationo et al. 2020, 2019; Greppi et al. 2017; Saubade et al. 2018) (Table 2). Greppi et al. (2017) isolated *Limosilactobacillus fermentum* (formerly known as *Lactobacillus fermentum* ) 8.2 and *L. plantarum* 6.2 from ben‐saalga. *L. plantarum* 6.2 produced more folate (4.4 μg/100 mL) in comparison to *L. fermentum* 8.2 (2.9 μg/100 mL). Bationo et al. (2019) also used *L. fermentum* 8.2 and *L. plantarum* 6.2, isolated from ben‐saalga to ferment pearl millet porridge. Spontaneous fermentation resulted in more folate (7.1–7.3 μg/100 g) than back‐slopping (6.1 μg/100 g). In another study, seven cereal‐based fermented foods were investigated for their folate content, including ben‐saalga. Although the folate content of pearl millet was high (54.4–73.4 μg/100 g), the folate content was low in the porridges (1.9–2.9 μg/100 g) (Bationo et al. 2020). The reduction in folate was attributed to grain processing steps, specifically debranning and degerming, rather than to fermentation itself, as the folate content of the dough made from non‐debranned grains remained appreciable following fermentation (Bationo et al. 2020). Similarly, low levels of folate (2.2 μg/100 g) were also observed by Saubade et al. (2018) in ben‐saalga.

In Sub‐Saharan Africa, different indigenous leafy vegetables are consumed in the daily diet. Misci et al. (2021) enhanced the nutritional quality of pumpkin leaves (*Cucurbita* sp.) by spontaneous fermentation. Sixty‐three different colonies of microorganisms were identified, and the most predominant was *Lactobacillaceae*. The folate content of fermented leaves (269.9 ± 8.3 μg/100 g) was significantly (*p* < 0.05) higher than that of the unfermented leaves (91.6 ± 7.1 μg/100 g) (Table 2).

A traditional Nigerian fermented cereal, Ogi, is prepared from different grains, including maize, millet, and sorghum (Figure 1C). Okoroafor et al. (2019) isolated LAB and yeast from maize slurry and then inoculated in the controlled fermentation of maize flour to prepare Ogi. Maximum folate concentration (3097 ± 0.37 μg/100 mL) was observed in the maize slurry fermented with *L. plantarum* X13 and *Candida tropicalis* Y74, which is three times more than the folate concentration of unfermented maize slurry. Researchers in another study isolated 43 strains of LAB from fermented locust beans, uncooked fermented cassava, cooked cassava (fufu), and cheese, and the highest amount of folate (24,105 μg/100 mL) was produced by *L. fermentum* MT903311 after 48 h on a vitamin assay (Odumosu et al. 2023).

Different species of yeasts isolated from cereals have also shown significant folate‐producing activity. In Tanzania, togwa, a maize‐based fermented porridge, is widely consumed in the rural areas. After 46 h of fermentation with *Candida glabrata* TY26, previously isolated from indigenous togwa, the folate concentration was found to be 6.9 μg/100 mL. This represents a remarkable 23‐fold increase in folate content compared to unfermented togwa (Hjortmo et al. 2008). Kariluoto et al. (2014) used previously isolated and identified yeast and bacterial strains to ferment oat and barley‐based cereal. The *S. cerevisiae* ALKO743 and *Candida milleri* ABM4949 were the high folate‐producing species. Net folate production was 12.0 μg/100 g at 24 h of fermentation (Table 2).

##### 3.2.1.2 Traditional Fermented Foods in Latin America

Tocosh is a famous fermented potato food of Peruvian origin and is prepared by the locals of the Central Peruvian Andes using traditional methods (Figure 1D). Researchers isolated several bacterial colonies from the tocosh samples. *Leuconostoc mesenteroides* and *Lactobacillus sakei* produced almost 7.0–10.0 μg/100 mL of folate (Jiménez et al. 2018). *L. sakei* CRL 2209 and CRL 2210 produced the highest concentrations of folate (76.0–148.4 μg/100 g). These strains were further utilized to ferment potato puree combined with amaranth and chia flour, which resulted in an 89%–95% increase in folate synthesis (Mosso et al. 2018; Table 2).

In Argentina, researchers isolated several LABs from quinoa and sourdough made through spontaneous fermentation. During fermentation, elevated folate concentrations were produced by *L. plantarum* CRL 1970, *L. rhamnosus* CRL 1972, and *L. sakei* CRL 1978. The highest concentration of folate in quinoa sourdough (14.3 ± 0.6 μg/100 mL) was produced by *L. plantarum* CRL 1973 (Carrizo et al. 2016). Later, the researchers used quinoa flour to make fermented pasta. The highest folate concentration (130.0 ± 10.0 μg/100 g) was achieved by *L. plantarum* CRL 2107 and *L. plantarum* CRL 1964 (Carrizo et al. 2020). Carrizo et al. (2017) also isolated *L. plantarum* species from spontaneously fermented amaranth sourdough. *L. plantarum* CRL 2107, which was previously identified from quinoa sourdough, along with *L. plantarum* CRL 2106, produced the highest amount of folate (13.8 ± 0.8 μg/100 mL). Another traditional fermented food from Argentina is chicha, which is a maize‐based fermented beverage. Rodrigo‐Torres et al. (2019) determined the concentration of folate biosynthesis by previously isolated strains of *L. plantarum* from chicha (Elizaquivel et al. 2015), which ranged between 3 and 5 μg/100 mL (Table 2).

##### 3.2.1.3 Traditional Fermented Foods in Asia

In China, fermented soybeans called *douchi* are very popularly consumed. Liu et al. (2019) isolated *L. plantarum* 4_3 from fermented soybean, and the maximum folate concentration (1.4–1.6 μg/100 mL) was observed at 24 h of fermentation (Table 2).

##### 3.2.1.4 Novel Fermented Foods of Plant Origin

In addition to studying traditionally fermented foods, researchers have increasingly explored the development of novel fermented products. In India, researchers fermented cucumber and watermelon juice with *Lactococcus lactis* subsp. *cremoris* isolated from raw cow milk. Net folate production in cucumber juice was 6.0 μg/100 mL, and in watermelon juice was 2.6 μg/100 mL (Gangadharan and Nampoothiri 2011). Apart from using microbial cultures isolated from the food, researchers have also utilized laboratory‐cultured or commercially available microbial strains. In a study, cauliflower and white beans were mixed and fermented using commercially available strains of *L. plantarum* (strain 299, 299v, Lp900, and Heal19). The highest folate concentration (58.8 ± 2.0 μg/100 g) was observed with *L. plantarum* 299v, which is 60% more than the initial value (Thompson et al. 2020). Albuquerque et al. (2017) fermented soymilk supplemented with passion fruit byproducts and fructo‐oligosaccharides. The maximum amount of folate (132.5 ± 7.7 μg/100 mL) was observed in soymilk with fructo‐oligosaccharides fermented by *Streptococcus thermophilus* ST‐M6, and the addition of passion fruit byproduct resulted in 125.0 ± 7.7 μg/100 mL folate (Albuquerque et al. 2017) (Table 2).

#### 3.2.2 Fermented Foods of Animal Origin

##### 3.2.2.1 Traditional Fermented Foods in Asia

**FIGURE 2:**
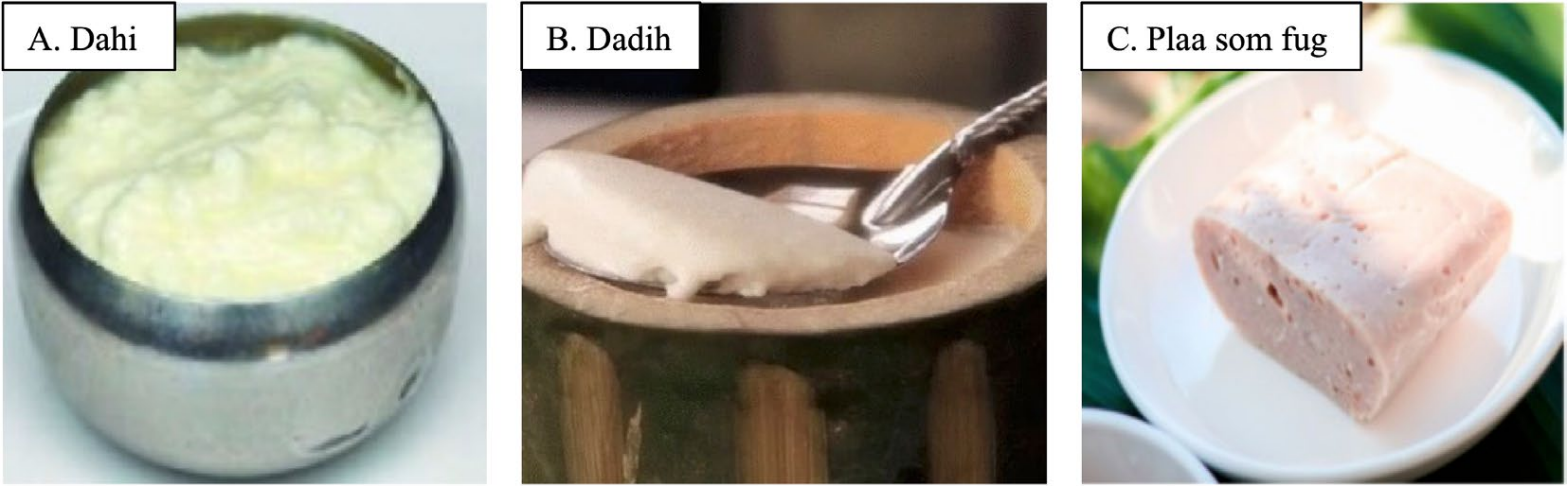
Fermented foods of animal origin: (A) Dahi [adapted from (Palika et al. 2020)], (B) Dadhi [adapted from (Herlina and Setiarto 2024)], (C) Plaa som fug [adapted from (Sivamaruthi et al. 2022)].

Yogurt is one of the most common fermented foods of animal origin consumed worldwide (Cuamatzin‐Garcia et al. 2022) and is known by different names across various regions. One such example is Dahi, a traditional, artisanal fermented milk product commonly consumed in the Indian subcontinent (Table 3; Figure 2A). *Enterococcus mundtii* QAUEM2808 was isolated from Dahi, and the *in silico* genome analysis suggested substantial folate production of approximately 2000–3000 μg/100 mL by the strain in culture medium (Nawaz et al. 2019). In West Sumatra, Indonesia, traditional fermented buffalo milk, Dadih, has been consumed for a very long time (Figure 2B). Seventeen bacterial strains isolated from Dadih produced folate in a range of 1.2 to 2.8 μg/100 mL. Out of these, 16 strains were identified as *L. plantarum* . The highest amount of folate (2.9 ± 0.4 μg/100 mL) was produced by *L. plantarum* Dad‐13 (Purwandhani et al. 2018). Apart from fermented dairy products, fermented meat is also consumed in some parts of the world. Famous fermented Thai fish, plaa som fug (Figure 2C), was analyzed for its microbial culture and the isolated *Weissella* spp. was studied further for folate production. Out of 11 strains of *Weissella cibria* and *W. confuse*, the PL12‐3 strain of the former species produced the maximum concentration (414 μg/100 mL) of folate (Deatraksa et al. 2018) (Table 3).

Zhang et al. (2020) isolated high folate‐producing strain *L. plantarum* GSLP‐7 V from Tibet kefir to prepare folate biofortified yogurt. The folate content of yogurt was substantially higher (372 μg/100 mL) compared to previous studies (Khalili et al. 2019; Laiño et al. 2019) (Table 3).

##### 3.2.2.2 Traditional Fermented Foods in Latin America

Laiño et al. (2012) evaluated Argentinian yogurt and reported on the folate‐producing capability of the native strain of *Lactobacillus delbrueckii* subsp. *bulgaricus* CRL 863 (Table 3). This strain, along with *S. thermophilus* was able to enhance the folate content of fermented milk by 190%. Later, the researchers prepared 15 separate samples of folate‐enriched yogurt using 5 distinct strains of the above‐mentioned microbial cultures and achieved maximum folate content with strains CRL871 + CRL803 + CRL415 (Laiño et al. 2013). This produced a much higher folate concentration as compared to their previous study (18.0 ± 1.0 μg/100 mL vs. 2.2–13.5 μg/100 mL) (Laiño et al. 2012). In a subsequent study, the authors discovered the significantly higher folate (81.2 ± 5.4 μg/L) producing capacity of *L. amylovorus* CRL887 among different lactobacilli. Yogurt produced from this strain with the starter culture selected from their previous study ( *L. bulgaricus* and *S. thermophilus* ) had sufficient folate to provide 16.4% of the RDA in a single 250 mL serving (Laiño et al. 2014; Table 3). Later, the researchers discovered the folate‐producing nature of *Streptococcus macedonicus* , and fermented milk was produced using a starter culture of *S. thermophilus* CRL803, *S. macedonicus* CRL415, and *L. bulgaricus* CRL871. This increased the folate concentration of milk to 18.7 ± 0.7 μg/100 mL as compared to the control fermented milk (7.5 ± 0.4 g/100 mL) (Laiño, Zelaya, et al. 2015). In another study, researchers further explored the folate‐producing characteristics of *S. macedonicus* CRL415. This bacterium alone was able to produce 4.3 ± 0.3 μg/100 mL folate (Laiño et al. 2019) (Table 3).

In another study, LAB were isolated from goat milk and cheese in Brazil to analyze the folate‐producing capability. Five strains of *S. thermophilus* (FP 34, 170, 268, 341, and 361) and two of *Lc. lactis* (FP343 and 368) were used to prepare fermented goat milk. These strains increased the folate concentration from 4.0 μg/100 mL to between 9.2 and 31.3 μg/100 mL. *Lc. lactis* FP368 fermented milk had 31.3 μg/100 mL folate, which is equivalent to 19% of the RDA of this vitamin for a 250 mL serving (Da Silva et al. 2016; Table 3).

##### 3.2.2.3 Novel Fermented Foods of Animal Origin

In addition to traditionally consumed fermented animal‐based food products, numerous studies have focused on fermenting milk using high folate‐producing bacterial strains isolated from dairy products. In India, researchers isolated LAB from raw cow milk and then biofortified skim milk with *Lc. lactis* CM28. The folate content in skim milk was 6.1 ± 1.3 μg/100 mL (Divya and Nampoothiri 2015b; Table 3). Divya and Nampoothiri (2015a) further isolated two LAB, *Lc. lactis* CM22 and *Lc. lactis* CM28 from cow milk, and prepared fermented skim milk and ice cream with these strains. Fermentation with *Lc. lactis* CM22 resulted in higher folate in both skim milk (14.4 ± 0.2 μg/100 mL) and ice cream (17.4 ± 0.1 μg/100 mL) compared to *Lc. lactis* CM28. Tidona et al. (2018) isolated *Lactococcus hircilactis* and *Lactococcus laudensis* from cow and goat milk from Italy and prepared fermented milk. *Lc. hircilactis* produced four times higher folate (1.6 ± 0.1 μg/100 mL) than the unfermented milk (Tidona et al. 2018; Table 3).

Some studies also utilized commercially available or lab‐grown microbial strains to ferment food products of animal origin. Jiao et al. (2020) prepared fermented milk using *Lc. lactis* KLDS4.0325 with the folate concentration of 60.0 μg/100 mL. In another study, a functional yogurt was prepared using *L. plantarum* , *L. casei* , and *L. acidophilus* . *L. plantarum* produced the highest folate, 6323 μg/100 mL (Wu et al. 2017; Table 3). To our knowledge, this is the highest concentration of folate ever recorded in a fermented food. In a similar study, researchers produced 5 different versions of folate biofortified yogurt using *L. bulgaricus* , *L. acidophilus* , *S. thermophilus* , *Bifidobacterium lactis* , *L. plantarum* 15HN, *L. plantarum* LAT BY PL, and *Lc. lactis*. Maximum folate concentration (148.7 ± 96.4 μg/100 mL) was achieved with *L. plantarum* 15HN (Khalili et al. 2019). In another study, *B. catenulatum* and *S. thermophilus* were inoculated in the milk, supplemented with prebiotics fructo‐oligosaccharides and galacto‐oligosaccharides; folate production was higher with *B. catenulatum* (28.8 ± 2.0 μg/100 mL) in the complex media, but in milk, *S. thermophilus* produced the highest amount of folate (19.0 ± 2.0 μg/100 mL) (Padalino et al. 2012). Fruit by‐products were also tested to enhance the folate content of fermented beverages. Cucick et al. (2024) prepared a folate‐enriched fermented whey beverage using grape, passion fruit, and dragon fruit (pitaya) by‐products. *B. infantis* BB‐02 and *S. thermophilus* TH‐4 produced the maximum amount of folate (40.7 μg/100 mL) with grape water extract (Table 3).

### 3.3 Animal Studies on Folate Bio‐Fortified Fermented Foods

Multiple studies have been conducted using an animal model to check the efficacy of folate biofortified foods. The most common method is the depletion‐repletion, where animals are depleted of folate for several weeks with a folate‐poor diet, followed by a repletion period with a diet containing the food to be studied (Table 4).

**TABLE 4:** Animal studies investigating folate bio‐fortified fermented foods.

Source of folate	Dose	Duration	Results	References
Fermented foods of plant origin				
Bio‐enriched fermented quinoa pasta ( *L. plantarum* )	Pasta containing 130 ± 10 μg/100 g of folate *ad libitum*	48 days of bio‐enriched diet	Increase in hematological values, whole blood and organ folate concentration, increase in the villi length of the small intestine	Carrizo et al. (2020)
Fermented foods of animal origin				
Bio‐enriched fermented milk (five *S. thermophilus* strains + *L. plantarum* 16cv)	50 mL of fermented milk containing 32.1 ± 1.4 μg/100 mL of folate	Depletion period: 14 days Repletion period: 21 days	Increase in hemoglobin, hematocrit and red blood cells and improvement in villi length and crypts depth	Cucick et al. (2020)
Biofortified yogurt ( *L. plantarum* GSLP‐7 V)	3 mL yogurt containing 372 μg/100 mL extracellular folate and 1202 μg/100 mL intracellular folate	Depletion period: 8 weeks Repletion period: 10 days	Increase in serum folate level, decrease in serum homocysteine level, alpha diversity of gut microbiota recovered	Zhang et al. (2020)
Folate enriched fermented milk (*Lc. lactis* subsp. *lactis* KLDS4.0325)	0.5 mL of fermented milk containing 60 μg/100 mL folate twice a day	Depletion period: 14 days Repletion period: 28 days	Increase in blood and liver folate and 5‐methyltetrahydrofolate concentrations, decrease in plasma homocysteine levels, improvement in gut microbiota composition	Jiao et al. (2020)
Fermented milk ( *L. bulgaricus* , *S. thermophilus* , and *S. macedonicus* )	Fermented milk containing 18.7 ± 0.7 μg/100 mL folate *ad libitum*	Depletion period: 14 days Repletion period: 21 days	Increase in plasma and whole blood folate concentrations, decrease in homocysteine levels	Laiño, Zelaya, et al. (2015)
Folate‐producing bacterial strains in the diet				
Lyophilized *L. plantarum* P2R3FA strain	Folic acid deficient diet supplemented with lyophilized *L. plantarum* P2R3FA strain containing 25.0 μg/100 g folate *ad libitum*	Depletion period: 30 days Repletion period: 28 days	Positive control exhibits more serum and erythrocytes folate than the strain group, strain group exhibit higher serum and erythrocyte folate than depleted group	Tamene, Baye, et al. (2019)
*Lc. lactis* NZ9000	Folic acid deficient diet supplemented with *Lc. lactis* strain containing 25.0 μg/100 g folate *ad libitum*	Depletion period: 30 days Repletion period: 28 days	Hematological value and morphology of blood cells returned to normal, serum and tissue folate concentration increased, no significant change in the erythrocyte folate concentration, lower plasma folate concentrations	LeBlanc et al. (2010)

#### 3.3.1 Fermented Foods of Plant Origin

Carrizo et al. (2020) studied bio‐enriched quinoa pasta fermented with *L. plantarum* in animal models (Table 4). There was no notable difference in the dietary intake between the control and experimental groups. Bio‐enriched pasta increased the folate levels of the whole blood, but there was no difference in the plasma folate concentration. Folate storage levels in the liver and spleen of the bio‐enriched diet group were higher than those of the other experimental groups. An increase in the villi length of the small intestine was also reported, which is important for the absorption of nutrients from the intestines.

#### 3.3.2 Fermented Foods of Animal Origin

Cucick et al. (2020) investigated the efficacy of bio‐enriched fermented milk prepared with *S. thermophilus and L. plantarum* (isolated from Brazilian goat dairy products) using a depletion‐repletion mice model (Table 4). After repletion, the animal's hemoglobin, hematocrit, and red blood cell levels increased. Their intestinal villi height to crypt depth ratio was comparable to that of animals receiving milk supplemented with synthetic folic acid. The bacterial strains used in fermentation demonstrated sensitivity to a wide range of antibiotics and lacked virulence genes, suggesting that the bio‐enriched fermented milk could serve as an alternative to synthetic folic acid.

In a similar study, Zhang et al. (2020) prepared a biofortified yogurt using *L. plantarum* isolated from Tibet kefir and studied its efficacy relative to supplemental folic acid in folate‐deficient rats. After repletion, the rats recovered the serum folate and homocysteine levels back to normal, but only the rats fed the biofortified yogurt improved their gut dysbacteriosis (imbalance of the gut microbiota (Petersen and Round 2014)), while the rats supplemented with folic acid worsened it (Table 4).

Jiao et al. (2020) compared folate‐enriched fermented milk to supplemental folic acid for their ability to replenish folate levels in folate‐depleted mice. Folate‐enriched fermented milk led to an increase in folate and 5‐methyltetrahydrofolate concentrations in whole blood and liver, while simultaneously decreasing plasma homocysteine levels. Folate‐enriched fermented milk alone or combined with folic acid significantly improved the gut microbiota composition compared to folic acid supplementation alone.

Another study assessed the efficacy of milk fermented with high folate‐producing strains of *L. bulgaricus* , *S. thermophilus* , and *S. macedonicus* . The mice that received fermented milk experienced a significant increase in plasma and whole blood folate concentrations and a decrease in homocysteine levels compared to the control group (Laiño, Zelaya, et al. 2015; Table 4).

#### 3.3.3 Folate‐Producing Bacterial Strains in the Diet

Alternatively, some studies have utilized high folate‐producing bacterial strains directly in the diet rather than the fermented products. Tamene, Baye, et al. (2019) used a high folate‐producing strain *L. plantarum* P2R3FA isolated from fermented tef dough in a folate depletion‐repletion model (Table 4). The experimental group was given a folate‐deficient diet supplemented with lyophilized *L. plantarum* P2R3FA strain. The strain was able to enhance serum and erythrocyte folate in the depleted rats, although to lower levels than in rats fed a normal diet. This was likely because the control group had a higher total dietary folate intake than the experimental group.

In a similar study, genetically engineered *Lc. lactis* strain providing 25.0 μg/100 g folate was used in a rodent depletion and repletion model (LeBlanc et al. 2010). Folate levels in the organs and blood increased significantly after repletion, while there was no difference in the erythrocyte folate concentration. The folate produced by this strain showed similar bioavailability to the commercially prepared folic acid (LeBlanc et al. 2010; Table 4).

## 4 Discussion

This review broadly summarizes the microbial strains present in different traditional fermented foods of animal and plant origin from various regions of the world, their folate concentration and their ability to improve folate status in animals. *L. plantarum* was found to be present in the foods with the highest folate concentration of both plant and animal origin. Nigerian ogi fermented with *L. plantarum* and *C. tropicalis* had the highest folate among plant‐based foods (3097 μg/100 mL) (Okoroafor et al. 2019), while yogurt containing *L. plantarum* had the highest folate in animal‐based foods (6323 μg/100 mL) (Wu et al. 2017).

Folate analysis is challenging due to the different forms of folate and their limited stability, which influence the capacity, performance, and specificity of analytical methods (Strandler et al. 2015). The studies included in this review used a range of methods to quantify folate in traditional fermented foods, including microbiological assay (*n* = 33), HPLC (*n* = 7), spectrophotometry (*n* = 2), and LC–MS/MS (*n* = 1), introducing methodological heterogeneity that should be considered when interpreting reported values. The microbiological assay, historically regarded as the reference method and the most frequently used approach in the included studies, measures total folate content but does not distinguish between individual folate forms unless complex differential assays are performed (Koontz et al. 2005). Therefore, comparison of specific folate forms across studies was not possible in this review. In addition, microbiological assays are susceptible to matrix effects from growth‐promoting or growth‐inhibiting compounds present in fermented foods, potentially leading to under‐ or overestimation of folate content (Koontz et al. 2005). Spectrophotometric methods also have limited specificity and are prone to interference from other compounds in food matrices, compromising folate quantification accuracy (Arcot and Shrestha 2005). HPLC offers better specificity by separating individual folate forms; however, it requires enzymatic deconjugation and careful sample handling to prevent folate degradation, which may otherwise result in underestimation (Doherty and Beecher 2003). LC–MS/MS provides the highest analytical sensitivity and specificity, enabling simultaneous quantification of multiple folate forms while minimizing matrix‐related interference, but its use remains limited because of its technical complexity and cost (Gebrehiwot et al. 2025). Therefore, direct comparisons of folate concentrations across studies should be made cautiously, as observed differences may reflect methodological variation as well as true biological differences.

Apart from the analytical methods, the data summarized in Tables 2 and 3 indicate that folate production is strongly influenced by microbial strain, food raw material and fermentation conditions. Considerable variation was observed even among strains belonging to the same species and mixed starter cultures frequently yielded higher folate concentrations than single‐strain fermentations, possibly because of synergistic metabolic interactions among microorganisms (Okoroafor et al. 2019). Among foods of plant origin, folate concentrations ranged from 2 to 200 μg/100 mL, with two notable exceptions reporting substantially higher values. One study reported a concentration of 3097 ± 37 μg/100 mL (Okoroafor et al. 2019), which may be attributed to the presence of yeast ( *C. tropicalis* ) in the microbial culture along with *L. plantarum*. Another study reported an exceptionally high value of 24,105 μg/100 mL (Odumosu et al. 2023), however, this value reflects folate production by the bacteria rather than the concentration present in fermented food. Fermentation conditions, particularly temperature (commonly reported in the range of 30°C–42°C) and incubation time (typically 24–48 h across the reviewed studies), further affected folate yield, indicating that process optimization is necessary to maximize folate production. For the foods of animal origin, reported folate concentrations typically ranged from approximately 0.1 to 300 μg/100 mL, with one study reporting a markedly higher concentration of 6323 μg/100 mL (Wu et al. 2017), which may be attributable to fermentation conditions, as folate was measured after 18 h of fermentation (Wu et al. 2017). Previous evidence suggests that folate concentrations can fluctuate during fermentation and that more stable levels are often observed after longer fermentation periods (e.g., 48 h) (Greppi et al. 2017), however, as the present review does not assess folate stability over time or systematically compare studies on this basis, further research is needed to address this limitation. Reported folate levels also differ depending on whether results are expressed on a dry weight or fresh weight basis, with higher concentrations generally observed in dry samples due to moisture removal (Greenfield and Southgate 2003; Singh and Heldman 2001). Due to these differences, direct comparison among studies remains challenging. Collectively, these findings indicate that folate concentration is influenced by strain characteristics, substrate composition, and fermentation conditions and these variations should be considered when interpreting and comparing folate concentration across studies.

Although bacterial cultures have been the primary focus of studies on folate biosynthesis in fermented foods, evidence from the present review indicates that yeasts may play an equally important role in folate production (Okoroafor et al. 2019). Several yeast species possess a high intrinsic capacity for folate synthesis and can contribute substantially to total folate levels, either independently or through metabolic interactions with bacteria in mixed cultures (Hjortmo et al. 2008) (Kariluoto et al. 2014). Similarly, emerging evidence highlights the folate‐producing potential of symbiotic cultures of bacteria and yeast (SCOBY), commonly used in the fermentation of kombucha and related milk‐ and plant‐based beverages (de Santana Khan et al. 2024; Frolova et al. 2023). These mixed microbial systems represent a promising approach for the development of novel fermented foods with enhanced folate content (Sanwal et al. 2025). Further research is required to explain the specific contributions of bacterial and yeast populations to folate biosynthesis during fermentation.

The animal studies included in this review provide preliminary evidence that folate‐biofortified fermented foods and folate‐producing bacterial strains can improve biomarkers of folate status in folate‐depleted models. Across the seven identified studies, consumption of folate‐enriched fermented foods, including quinoa pasta (Carrizo et al. 2020), fermented milk (Cucick et al. 2020; Jiao et al. 2020; Laiño, Zelaya, et al. 2015), and yogurt (Zhang et al. 2020), generally increased folate concentrations in blood and tissues. Despite these promising findings, the evidence base remains limited and only seven animal studies were identified, most using depletion–repletion models under controlled experimental conditions. While these models are useful for assessing folate efficacy, they may not accurately reflect the complexity of dietary intake patterns, folate bioavailability, and metabolism in humans. Human studies examining folate bioavailability from fermented foods have also reported considerable inter‐individual variability (Obermaier et al. 2024), emphasizing the need for well‐controlled large‐scale studies into limiting factors such as food matrix, microbial strains, and fermentation conditions (Keyvan et al. 2025). Therefore, the translational relevance of the findings from existing animal studies to human populations remains uncertain, and confirmation through well‐designed clinical studies is required.

### 4.1 Limitations

This review has several limitations that should be considered when interpreting the findings. First, the literature search was restricted to a single database (PubMed), which may have resulted in the exclusion of some relevant studies indexed elsewhere. Second, substantial heterogeneity exists among the included studies with respect to food matrices, microbial strains, fermentation conditions, sample preparation procedures, and folate quantification techniques, making direct comparisons challenging. These variations may also contribute to the wide range of folate concentrations reported for similar fermented foods. Therefore, the findings presented in this review should be interpreted with caution, and the reported folate values should be considered within the context of the specific methodologies used in each study. Third, the animal studies included in this review exhibit significant variability in terms of microbial species, fermentation processes, folate doses, trial durations, and outcome measurements. This heterogeneity limits direct comparisons across the studies and reduces their translational relevance to humans.

## 5 Conclusion and Future Developments

Both plant‐based and animal‐based traditional fermented foods can contain appreciable amounts of folate and could be a promising dietary source of this vitamin. Several bacterial species exhibit folate‐producing potential that could potentially increase the folate concentration of fermented foods, and genetic modifications may further enhance the folate‐producing capacity of the bacterial strain. Evidence from the limited number of available animal studies suggests that consumption of folate‐enriched fermented foods may improve folate status under experimental conditions.

To fully evaluate the potential of fermented foods in improving dietary folate intake, further research focusing on traditional high folate fermented foods, identifying high folate‐producing microbial strains suitable for food biofortification, and evaluating their probiotic and folate‐enhancing potential in animal models and human trials is needed. These efforts could help develop accessible and culturally appropriate dietary strategies to address folate deficiency.

## Author Contributions

**Sanaullah Iqbal:** conceptualization, methodology, data curation, formal analysis, supervision, visualization, writing – original draft. **Rida Khan:** conceptualization, methodology, data curation, formal analysis, visualization, writing – original draft. **L. Suzanne Suggs:** conceptualization, methodology, formal analysis, supervision, visualization, writing – review and editing. **Pedro Marques‐Vidal:** conceptualization, methodology, formal analysis, supervision, visualization, writing – review and editing.

## Funding

This project has received funding from the Swiss Government Excellence Scholarship No. 2024.0443.

## Conflicts of Interest

The authors declare no conflicts of interest.

## Data Availability

No new datasets were generated during this study. All data supporting the findings of this review are available in the published articles included in the review, which are cited in the reference list with their respective DOIs.
